# Effect of contaminations and surface preparation on the work function of single layer MoS_2_

**DOI:** 10.3762/bjnano.5.32

**Published:** 2014-03-13

**Authors:** Oliver Ochedowski, Kolyo Marinov, Nils Scheuschner, Artur Poloczek, Benedict Kleine Bussmann, Janina Maultzsch, Marika Schleberger

**Affiliations:** 1 Fakultät für Physik and CeNIDE, Universität Duisburg-Essen, Lotharstr. 1, 47057 Duisburg, Germany; 2 Institut für Festkörperphysik, Technische Universität Berlin, Hardenbergstr. 36, 10623 Berlin, Germany; 3 Solid State Electronics Department and CeNIDE, University of Duisburg-Essen, Lotharstr. 55, 47058 Duisburg, Germany

**Keywords:** KPFM, MoS_2_, NC-AFM, surface potential, work function

## Abstract

Thinning out MoS_2_ crystals to atomically thin layers results in the transition from an indirect to a direct bandgap material. This makes single layer MoS_2_ an exciting new material for electronic devices. In MoS_2_ devices it has been observed that the choice of materials, in particular for contact and gate, is crucial for their performance. This makes it very important to study the interaction between ultrathin MoS_2_ layers and materials employed in electronic devices in order to optimize their performance. In this work we used NC-AFM in combination with quantitative KPFM to study the influence of the substrate material and the processing on single layer MoS_2_ during device fabrication. We find a strong influence of contaminations caused by the processing on the surface potential of MoS_2_. It is shown that the charge transfer from the substrate is able to change the work function of MoS_2_ by about 40 meV. Our findings suggest two things. First, the necessity to properly clean devices after processing as contaminations have a great impact on the surface potential. Second, that by choosing appropriate materials the work function can be modified to reduce contact resistance.

## Introduction

Due to their unique properties which can differ a lot compared to bulk materials, two-dimensional materials are being targeted in a variety of research areas like surface physics, electrical engineering, chemistry and biomedical applications [1–4]. The 2D-material getting the most attention besides graphene are single layers of molybdenum disulfide (SLM) which consist of a plane of molybdenum atoms that are sandwiched between sulfur atoms. The main reason for this is the transition from an indirect (bulk MoS_2_) to a direct (single layer MoS_2_) band gap semi-conductor [5]. Single layer MoS_2_ has a strong photoluminescence signal [5–9] and other interesting properties like a mechanical stiffness of 180 ± 60 N·m^−1^, which is comparable to steel [10–11], charge carrier mobilities that are comparable to Si [12–13], and it is possible to grow these ultrathin layers using CVD [14–16]. The main advantage SLM has to offer compared to the model 2D-material graphene is its direct band gap. It allows the facile integration of SLM in electronic devices, which has been demonstrated for highly flexible transistors, optoelectronic devices, small-signal amplifiers, MoS_2_ integrated circuits and chemical vapor sensors [12,17–21]. It has been reported that the performance of these devices can greatly vary due to the choice of the material of the contacts, the cleanliness of the SLM surface and a top gated structure with a high *κ* dielectric [22–27]. By choosing appropriate materials in 2D-devices the work function can be tuned to, e.g., lower the contact resistance and improve their performance. First experiments adressing this issue for MoS_2_ by using Kelvin probe force microscopy (KPFM) have already been reported [28–29]. However, these measurements were not done on SLM but bilayer MoS_2_ (BLM) and higher layer numbers and the measurements were performed under ambient conditions using amplitude modulated KPFM, both having a great impact on the results. In this work we study the work function of SLM on a standard SiO_2_/Si substrate using non-contact atomic force microscopy (NC-AFM) and Kelvin probe force microscopy in situ. In our measurements we use a gold contact patterned on SLM in order to calibrate the work function of our AFM tip which allows us to determine quantitative work function values for SLM, BLM and few layer MoS_2_ (FLM). Additionaly, we use reactive ion etching to pattern holes into the SiO_2_ substrate. By comparing the work function of SLM on etched and pristine SiO_2_ substrates, we show that a significant change in the work function can be achieved by substrate effects.

## Experimental

**Figure 1:**
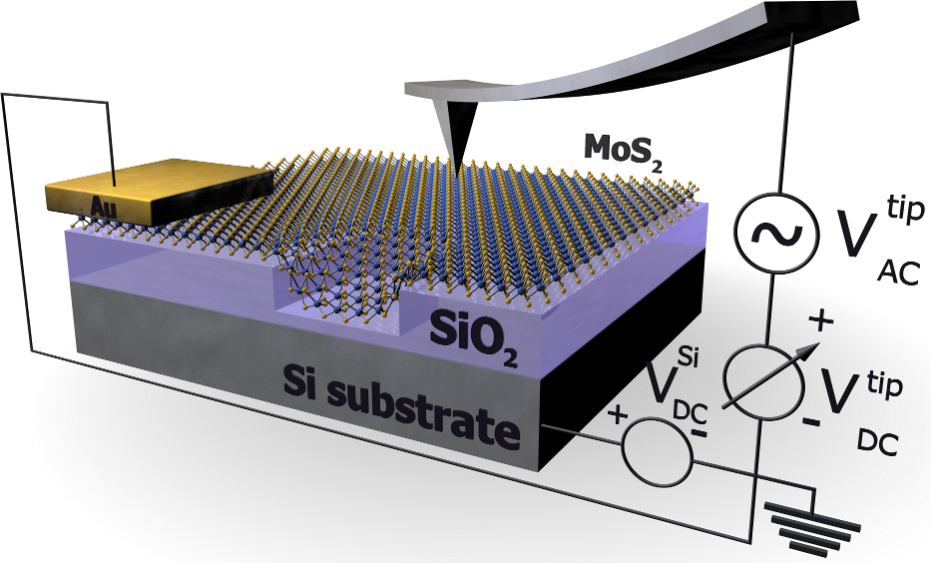
Schematic representation of the KPFM setup and the MoS_2_ sample with the RIE SiO_2_.

For our studies we exfoliated MoS_2_ (HQgraphene, Netherlands) on a patterned Si sample that has been covered by 90 nm SiO_2_ layer (graphene supermarket, Calverton, NY, USA). The SiO_2_ was patterned by using an inductive coupled plasma reactive ion etching (ICP-RIE) with Cl_2_/N_2_ chemistry. The etching mask used was a standard photoresist patterned by optical lithography. The etching was performed at 35 °C using 300 W of ICP and 150 W table power. The chamber pressure was adjusted to 8·10^−3^ mbar during this procedure. Reactive ion etching was employed to locally alter the surface roughness and introduce defects in the SiO_2_ substrate [30–31]. The resulting structures on the SiO_2_ surface consist of etched holes with a depth of about 40 nm measured using AFM. Immediately after etching, the MoS_2_ was exfoliated by mechanical cleavage [32]. Single layer MoS_2_ flakes were located by using their optical contrast and verified using Raman spectroscopy [33–34]. For Raman point measurements and mappings, a Renishaw InVia Raman spectrometer (λ = 532 nm, *P* < 0.4 mW, spectral resolution ≈ 1 cm^−1^) has been employed. Because SLM is highly flexibel, it is not covering the etched hole. Instead the SLM touches the etched SiO_2_ surface at the bottom and follows the morphology like a membrane (Figure 1). While this leaves the SLM heavily strained on the edge of the hole, it allows to experimentally compare the effect of two differently treated subtrates (SiO_2_ and RIE SiO_2_) on the same MoS_2_ flake. After identification of SLM areas, a Ti/Au (5 nm/15 nm) contact was patterned on the MoS_2_ flake by photolithography. We used the Photoresist ARP-5350 (Allresist GmbH, Strausberg, Germany) with the developer AR 300-35 (Allresist GmbH, Strausberg, Germany). Acetone was used for the lift-off and finally the samples were boiled in isopropyl alcohole. The contact served two purposes. On the one hand, the sample was electrically connected to ground potential, on the other hand, the gold surface was used for calibrating the work function of the AFM tip during KPFM measurements.

The contacted SLM sample is introduced into an ultra high vacuum system with a base pressure of about 2·10^−10^ mbar. Non-contact AFM measurements were performed using a RHK UHV 7500 system with the PLL Pro 2 controller. Simultaneously to NC-AFM, frequency-modulated KPFM measurements were conducted to probe the local contact potential difference (CPD) between the tip and the surface [35–41]. As force sensors, highly conductive Si cantilevers with a typical resonance frequency of *f* = 300 kHz (Vistaprobe T300) were utilized. During KPFM measurements an AC voltage is applied to the tip (*U*_AC_ = 1 V and *f*_AC_ = 1 kHz) and the built in lock-in amplifier of the PLL Pro 2 is used to apply a DC voltage which minimizes the resulting electrostatic forces between tip and sample surface. This DC voltage corresponds to the local CPD.

## Results and Discussion

### Raman spectroscopy characterization

**Figure 2:**
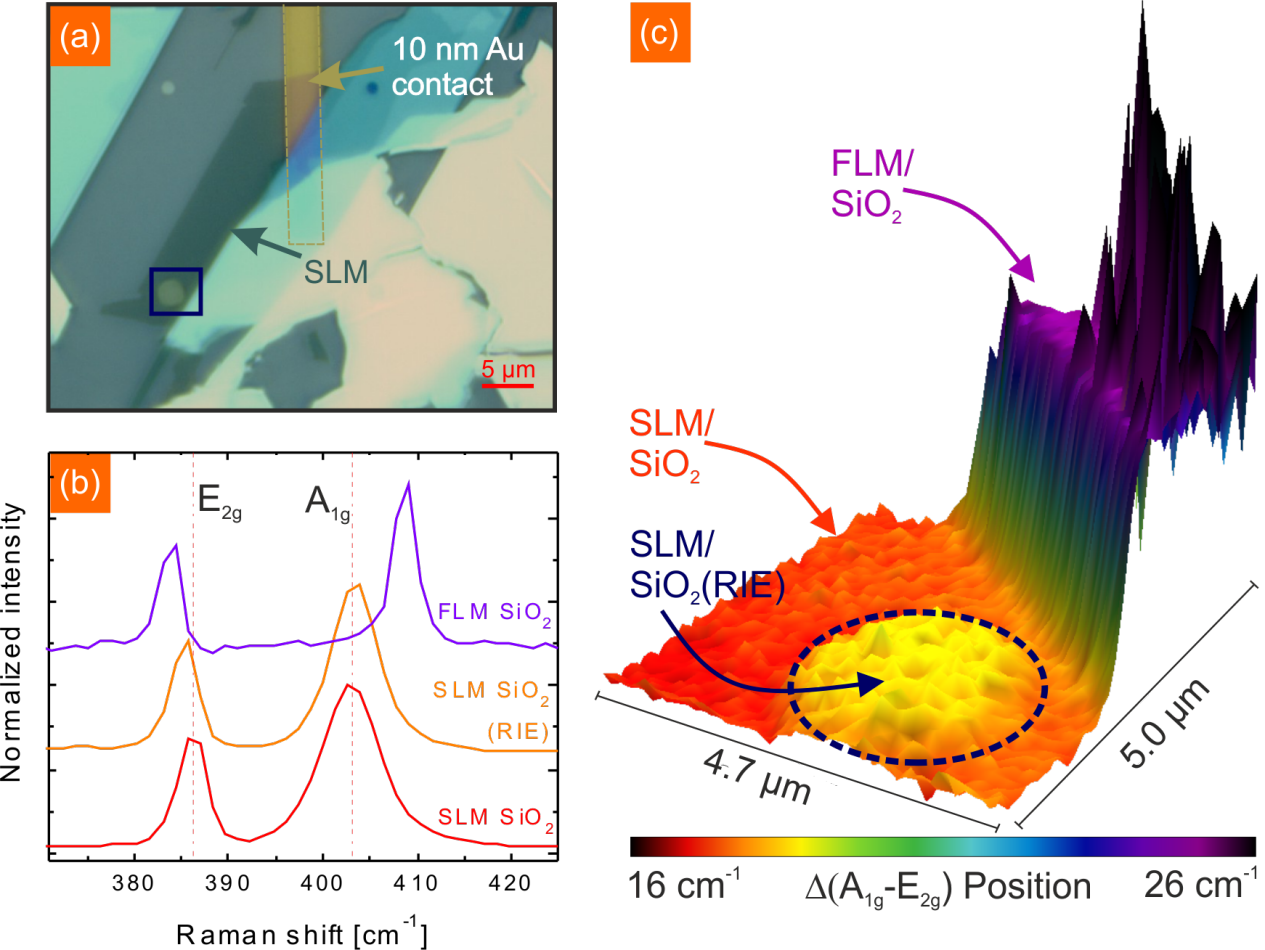
(a) Optical microscope image of an exfoliated MoS_2_ flake on a prepatterned (RIE) SiO_2_ substrate. A gold contact was attached to the MoS_2_ in order to ground the flake for KPFM measurements. (b) Raman spectroscopy spectra of SL and FL MoS_2_ on SiO_2_ and SL MoS_2_ on RIE SiO_2_. For higher layer numbers the *E*_2_*_g_* is shifted to lower wave number while the *A*_1_*_g_* mode is shifted to higher wave numbers. (c) Raman mapping data of the area marked in (a) with the blue box. The difference between *A*_1_*_g_* and *E*_2_*_g_* mode is plotted revealing a shift of the Raman modes for SLM on the RIE SiO_2_ substrate.

In Figure 2 we present an optical image of a sample prepared by the procedure described above together with additional Raman spectroscopy data. The SLM flake can be identified in the optical image in Figure 2a by its contrast, which is a transparent green tone. While the majority of the SLM flake is located on pristine SiO_2_, a small part of the SLM flake is at the bottom of a hole which was patterned by RIE. To unambiguously identify SLM we used Raman spectroscopy and compared the results to data obtained by literature [34]. In Figure 2b the Raman spectra of SLM on SiO_2_ and on SiO_2_ (RIE) as well as FLM on SiO_2_ is shown. The two prominent peaks, the *E*_2_*_g_* and *A*_1_*_g_* peak, correspond to the opposite vibration of the two S atoms with respect to the Mo atom and the out-of-plane vibration of only S atoms in opposite directions, respectively [42–43]. For SLM on SiO_2_ the Raman shifts obtained for the *E*_2_*_g_*, ν = 386.1 cm^−1^, and *A*_1_*_g_*, ν = 403.0 cm^−1^, are consistent with values reported by other groups. For higher layer numbers the *E*_2_*_g_* has been reported to shift to lower wave numbers while the *A*_1_*_g_* shifts to larger wave numbers which is again in good agreement with our data. However, the SLM on RIE SiO_2_ shows a different behaviour compared to SLM on pristine SiO_2_. The *E*_2_*_g_* is slightly downshifted to ν = 385.2 cm^−1^ and the *A*_1_*_g_* shows a minor shift to ν = 403.4 cm^−1^. Shifts of the *E*_2_*_g_* and *A*_1_*_g_* modes of SLM can have multiple reasons. Uniaxial tensial strain has been observed to cause a splitting in the *E*_2_*_g_* mode and a shift to lower wave numbers for the resulting *E*^−^ and *E*^+^ modes by 4.5 and 1 cm^−1^/% [44–45]. While the *A*_1_*_g_* mode shows no distinct sensitivity to uniaxial strain, a charge carrier dependency has been observed [46]. Electron doping of 1.8·10^13^ cm^−2^ leads to a linewidth broadening of 6 cm^−1^ and the phonon frequency decreases by 4 cm^−1^. As our data shows a shift in both Raman active modes we suggest that the RIE SiO_2_ surface causes a slight strain and maybe local doping by charge transfer in the MoS_2_ flake. The Raman mapping shown in Figure 2c corresponds to the evaluation of point spectra performed in the green box marked in Figure 2a. Plotted is the difference of the *E*_2_*_g_* an *A*_1_*_g_* mode positions. While the difference between SLM and FLM on SiO_2_ is significant with Δ = 8.2 cm^−1^, the difference between SLM on SiO_2_ and on RIE SiO_2_ is relatively small with Δ = 1.3 cm^−1^. As can be seen in the Raman mapping, the difference in the SLM induced by the substrate is constant over the whole flake and not just present in single point meaurements.

### In-situ KPFM on single layers of MoS_2_

**Figure 3:**
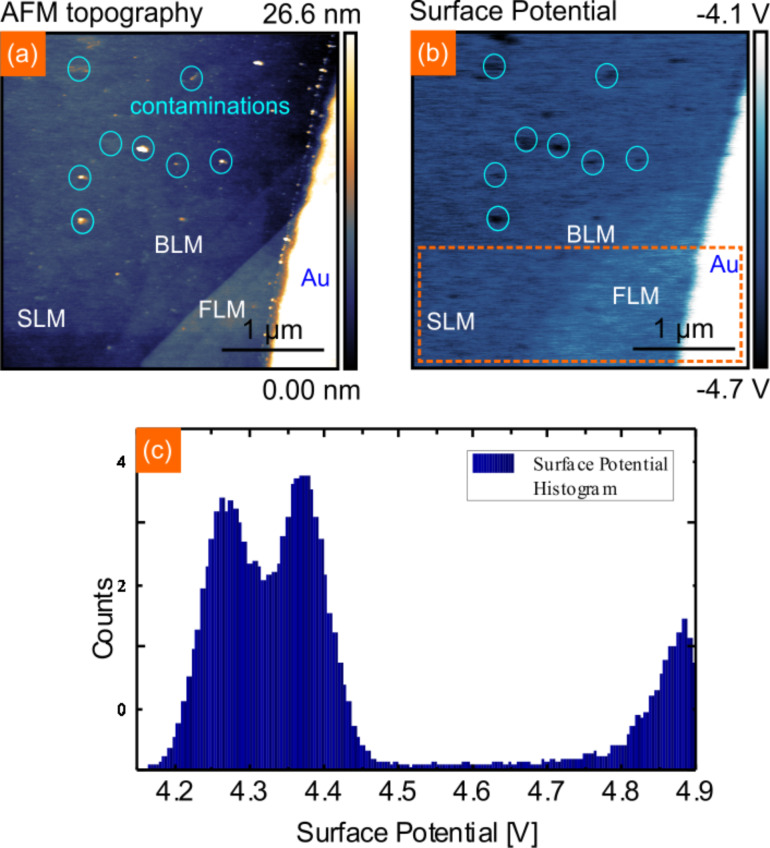
(a) NC-AFM image of MoS_2_ flake on SiO_2_ with a gold contact (height = 20 nm). Topography shows areas with contaminations due to processing. (b) Corresponding surface potential image to (a). The surface potential of MoS_2_ is increasing with increasing layer thickness, contaminations can be clearly distinguished in the surface potential image. (c) Surface potential histogram of the box marked in (b).

For the NC-AFM and KPFM measurements the sample was introduced to the UHV system. Before the data collection the sample was heated in situ to 200 °C for 30 min to remove any adsorbates from ambience. In Figure 3a and Figure 3c the NC-AFM topography and the corresponding surface potential map are shown, respectively. On the right side the Ti/Au contact can be seen which is about 20 nm high and shows a distinct contrast in the surface potential in comparison to the MoS_2_ layers. In Figure 3d a surface potential histogram of SLM, FLM and the gold surface of the Ti/Au contact is given. We find a surface potential of 4.27 V for SLM, 4.37 V for FLM and 4.89 V for gold. The surface potential itself is always a relative value based on the local CPD between the AFM tip and the sample surface. To obtain quantitative work function values, we calibrated the tip on the gold surface by using the known work function of gold Φ_Au_ = 5.10 eV [47–48]. With the relation Φ = 5.10eV − *e*·(CPD_Au_ − CPD_nMoS2_) the work function of SLM Φ_SLM_ = 4.49 ± 0.03 eV and FLM Φ_FLM_ = 4.59 ± 0.03 eV can be assigned. The given errorbar consists of the experimental error of our system. Not included in this error is band bending, which occurs when doing KPFM measurements on a semi-conductor surface and a false estimation of the work function of the patterned gold contact. Besides graphite [49], gold is a common material to calibrate the work function of the AFM tip [48], but while the work function Φ_Au_ = 5.10 eV is often used, other work function values in the range from 4.74 eV to 5.54 eV have been reported as well [50–51]. Surface roughness, homogeneity and humidity can have an effect on the measured work function of metal surfaces as Guo et al. recently demonstrated [52]. The presented data is measured in situ after annealing and we are therefore confident that humidity can be neglected. We want to point out that an error in the work function calibration does not affect the work function values of SLM, BLM and FLM with respect to each other. While the surface potential on the Au contact in Figure 3 appears uniform, strong local variations can be observed on the MoS_2_ flake. We attribute these features, marked in Figure 3a with green circles, to contaminations due to the patterning process. The height of these contaminations varies between 1 nm and 20 nm. These contaminations have a noticeable effect on the work function of SLM, as Φ_SLM_ can be lowered by up to 0.15 eV. As the work function of these contaminations is clearly different than that of the Au contact, the contaminations are most likely resist residues which have not been completely removed. Such contaminations may act as scattering centers or charge puddles which are likely to be detrimental to the performance of SLM devices [53]. For graphene and MoS_2_ it has been shown, that adsorbates due to ambient exposure can have a strong impact on the work function of these materials, like inducing an additional charge transfer or even redox reactions with water [29,54].

### In situ screening length of MoS_2_

**Figure 4:**
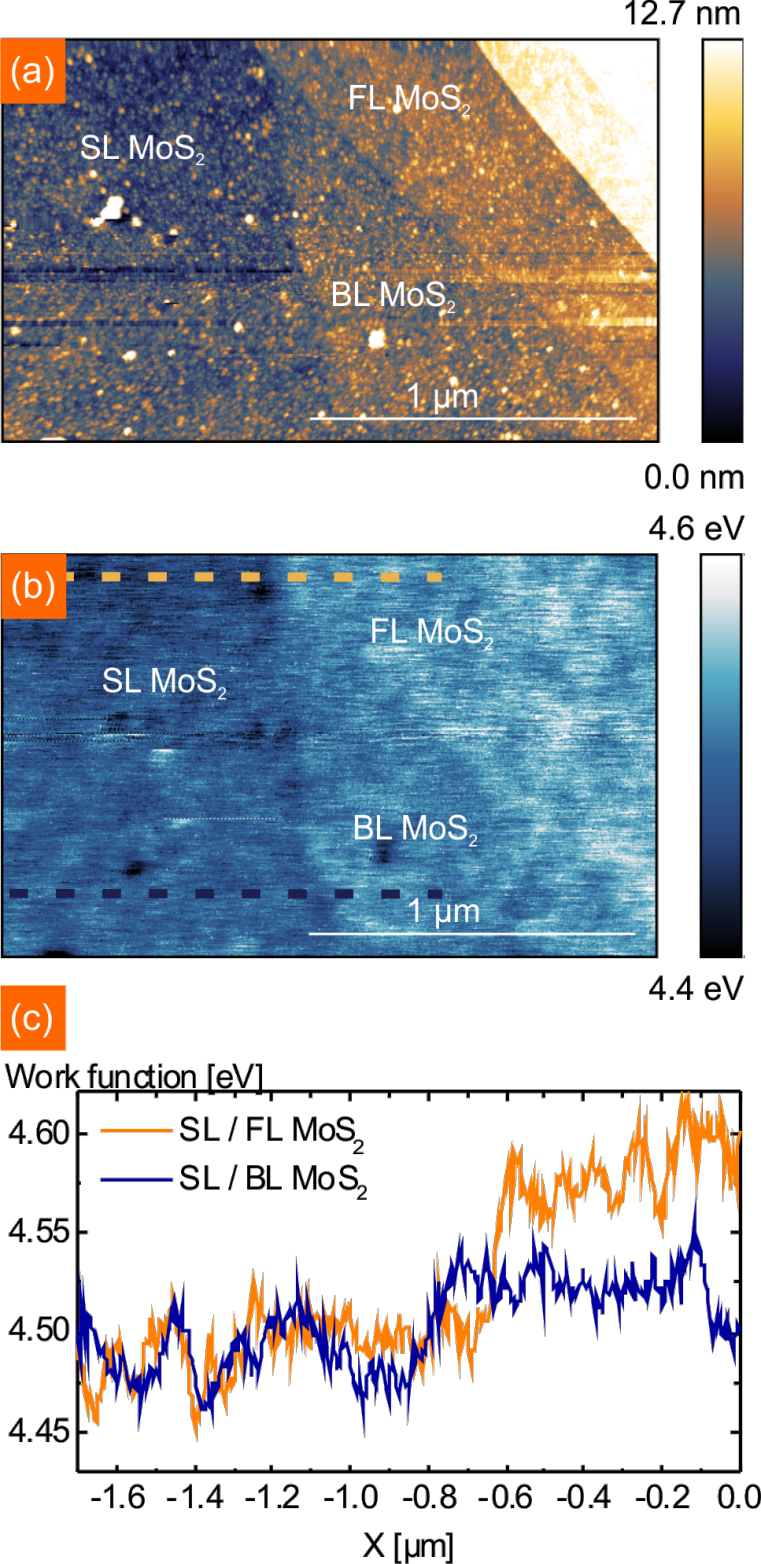
(a) NC-AFM zoom-in of an area consisting of 1L, 2L and FL MoS_2_. (b) Corresponding KPFM image, calibration of the tip on the gold contact allows assignment of work functions to surface potential values. Plotted is the work function. (c) Line profiles of the work function corresponding to the lines marked in (b).

In the next step, we determine the work function of BLM and the screening length of MoS_2_. For this the SLM/BLM/FLM section of Figure 3 has been measured again in more detail and the work function is analyzed by line profiles. Shown in Figure 4a–c are the NC-AFM topography, work function map and the corresponding line profiles, respectively. The measured height for BLM is 0.92 ± 0.10 nm, which is slightly higher than the interlayer spacing of a bulk MoS_2_ crystal [55]. For FLM we get two different heights, one is 2.96 nm (≈5 layers) and 7.89 nm (≈12–13 layers). In the work function map in Figure 4b, three contrasts can be observed – SLM, BLM and FLM. As the work function for FLM 2.96 nm and the other FLM with 7.89 nm is not changing, we conclude from our data that the screening length of MoS_2_ is at least 2.96 nm, which is in good agreement with previous findings for annealed MoS_2_ [29]. Li et al. compared the screening length of pristine MoS_2_ flakes on SiO_2_ with annealed MoS_2_ flakes and found a decrease from approximately 5 nm down to 2.5 nm for annealed MoS_2_. Our measurements here yield a screening length between 1.6 and 2.96 nm, which is much lower than the value for pristine MoS_2_. We therefore conclude that the investigated MoS_2_ is not affected by ambient adsorbates. In Figure 4c we used the line profile to quantify the work function of SLM and BLM. The work function of SLM is determined to be the same as using the histogram analysis in Figure 3 with Φ_SLM_ = 4.49 ± 0.03 eV. The work function of BLM is increased with respect to SLM by about 0.05 eV to Φ_BLM_ = 4.54 ± 0.03 eV. Again, contaminations on BLM appear to decrease the work function as can be seen in Figure 4b.

### Substrate effects on the work function of single layer MoS_2_

**Figure 5:**
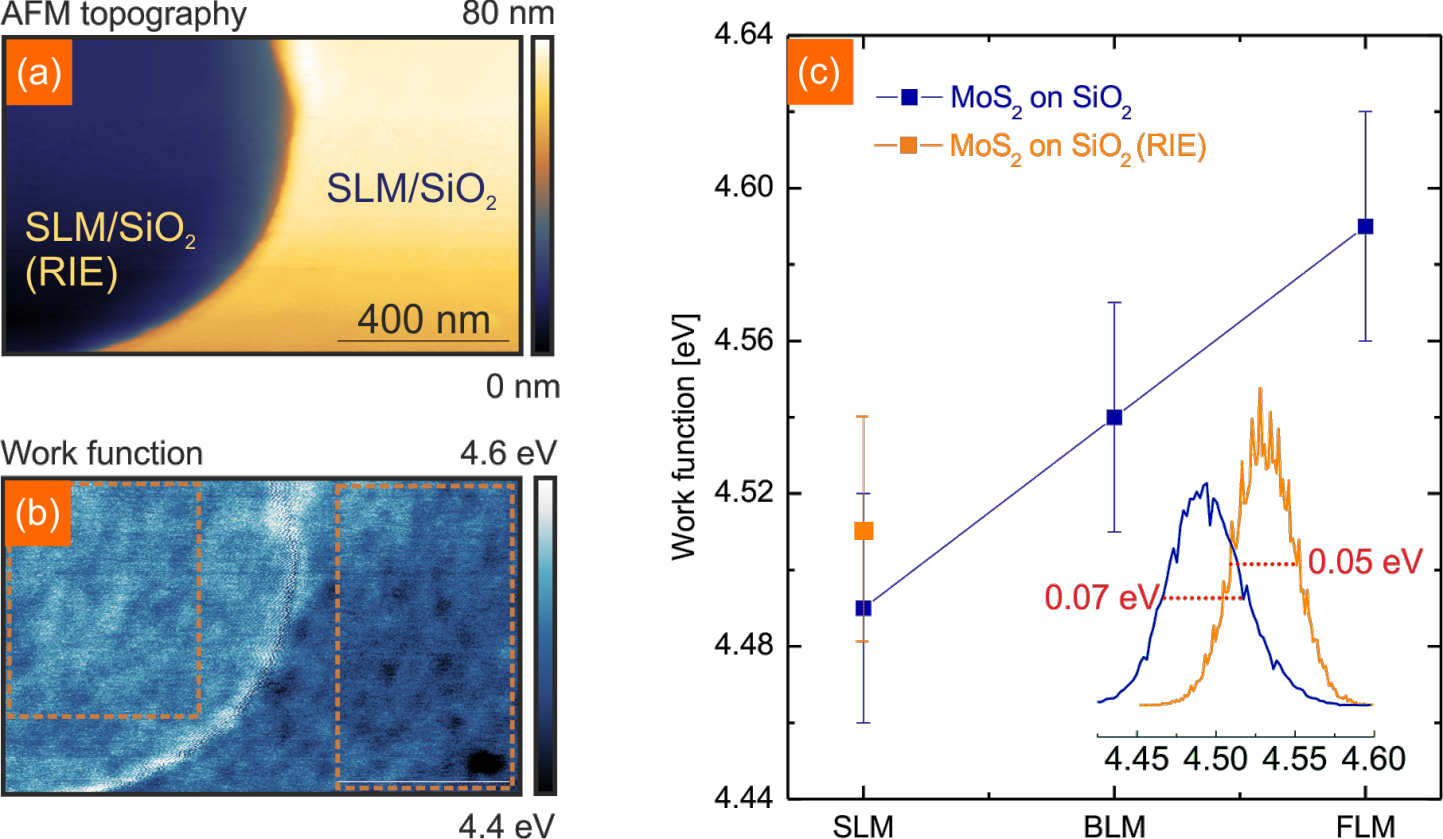
(a) NC-AFM topography of SLM on SiO_2_ and holes etched in SiO_2_ using RIE. (b) Work function map corresponding to the topography shown in (a). The work function of SLM on etched SiO_2_ is increased compared to pristine SiO_2_. (c) Layer dependent work function of MoS_2_. The inset shows the work function histogram evaluation of the areas marked in (b). The FWHM of SLM on RIE SiO_2_ is decreased by 0.02 eV.

To study the effect of the substrate on the work function of SLM, we compare the work function of SLM on SiO_2_ with SLM in the RIE SiO_2_ holes in Figure 5. The work function map in Figure 5b shows an increased work function over the etched hole of about ΔΦ = 0.04 eV. This shift is caused by the charge transfer from the etched substrate which leads to an effective doping that has been proven to have a large impact on the optical properties of SLM [56]. The etched SiO_2_ substrate has an effect on the surface potential distribution as well. By comparing histogram data of SLM on SiO_2_ and RIE SiO_2_ (see inset in Figure 5c) we find a decreased surface potential fluctuation by 0.02 eV for SLM on the etched SiO_2_. The potential fluctuation is related to charge impurities which are detrimental for the performance of 2D-devices and KPFM is an efficient way to probe it [57]. Further, a lower potential fluctuation indicates a higher charge homogeneity. Charge inhomogeneity has been shown to play a crucial role in the oxidative reactivity of graphene [58]. At the edge of the etched hole, where SLM is heavily bent, a strong increase in the work function by another ΔΦ = 0.05 eV compared to SLM on the RIE SiO_2_ substrate caused by stress can be observed. It has been shown by Castellanos-Gomez et al. that heavy strain in SLM has a large impact on the band gap of SLM [59]. However, KPFM only measures the contact potential difference (from which we derive the work function). For insulating materials there is no straightforward relation between the contact potential difference and the band-gap. Therefore, our results are not directly comparable. The plot in Figure 5c sums up our findings with respect to the work function of MoS_2_. The work function of FLM in ambient has been determined previously by amplitude modulated KPFM. The reported values of Φ = 5.25 eV [28] are significantly higher than the values found here. This difference is clearly due to the contaminations which are absent in our measurements. Our data should instead be compared to the values determined by other means like ultraviolet photoelectron spectrosocopy [60–63]. The excellent agreement again underlines the importance of UHV measurements if intrinsic properties are to be probed.

## Conclusion

In conclusion we have performed the first in situ Kelvin probe force microscopy measurements on single layers of MoS_2_ on a SiO_2_ substrate. We find work functions of Φ_SLM_ = 4.49 eV, Φ_BLM_ = 4.54 eV and Φ_FLM_ = 4.59 eV for SLM, BLM and FLM respectively. We observe a screening length between 1.6 and 3.5 nm which indicates a clean MoS_2_ flake. We have further investigated the effect of the substrate on the work function of MoS_2_ by partly etching the SiO_2_ substrate. Raman spectroscopy measurements suggests substrate effects like strain which increase the work function of SLM of ΔΦ = 0.04 eV on etched SiO_2_. The next step is to investigate completely free standing MoS_2_ flakes without a substrate in order to probe the intrinsic charge homogeneity and work function of SLM.
